# An epigenetic mechanism of azole tolerance facilitates acquired antifungal resistance in *Aspergillus fumigatus*

**DOI:** 10.1128/mbio.00661-26

**Published:** 2026-06-15

**Authors:** Sandeep Vellanki, Nathan DeMichaelis, Catherine Liao, Jason E. Stajich, Robert A. Cramer

**Affiliations:** 1 Department of Microbiology and Immunology, Geisel School of Medicine at Dartmouth, Hanover, New Hampshire, USA; 2 Department of Microbiology and Plant Pathology, University of California—Riverside 8790, Riverside, California, USA; University of Wisconsin-Madison, Madison, Wisconsin, USA

**Keywords:** *Aspergillus fumigatus*, antifungal drugs, azole resistance, drug tolerance

## Abstract

Antibiotic tolerance paves the way for acquired resistance in bacterial pathogens. However, the mechanisms of tolerance and its evolutionary role in acquired resistance in pathogenic fungi, and particularly in filamentous fungi, remain elusive. Here, we identified an Inhibitor of Growth domain-containing protein (IngB) as a novel epigenetic regulator of azole tolerance in *Aspergillus fumigatus*. The loss of *ingB* promotes supra-MIC growth on agar surfaces despite susceptible MICs in standardized assays. Moreover, established Δ*ingB* biofilms are also less susceptible to azoles *in vitro*. In a murine model of invasive pulmonary aspergillosis, loss of *ingB* results in higher pulmonary fungal levels when animals are treated with voriconazole compared to the wild-type control. Subsequent exposure of the Δ*ingB*-tolerant strain to high azole concentrations *in vitro* resulted in rapid acquired resistance, most notably driven by a frameshift mutation in a putative 20S proteasome maturation protein-encoding gene, *ump*A, while the susceptible wild-type strain failed to acquire adaptive mutations. The data suggest that loss of IngB provides an epistatic background for the emergence of azole resistance. Our work shows that drug tolerance in a critical fungal pathogen can facilitate azole resistance emergence.

**IMPORTANCE:**

While antimicrobial drug resistance causes adverse effects on human health, drug tolerance can also lead to insufficient pathogen clearance, resulting in infection relapse. However, the mechanisms of antifungal drug tolerance and its evolutionary role in acquired drug resistance in pathogenic fungi, particularly the molds, remain elusive. We identified IngB as a novel regulator of azole tolerance in *Aspergillus fumigatus*. In a murine model of invasive pulmonary aspergillosis treated with voriconazole, loss of *ingB* facilitated higher fungal burden levels than the wild-type control, suggesting the observed *in vitro* tolerance translates to the murine pulmonary environment. Importantly, loss of IngB leads to rapid azole drug resistance under azole-selective pressure *in vitro* and led to the discovery of a new gene associated with azole resistance, *umpA*. Our work identifies a novel regulator of antifungal tolerance in a critical human fungal pathogen and suggests that drug tolerance can pave the way for resistance emergence.

## INTRODUCTION

Drug resistance is a primary global reason for clinical therapeutic failures, leading to prolonged illness, deaths, and higher hospitalization costs (1). Often discussed in the context of cancer or bacterial infections, the continually emerging resistance to pathogenic fungi also poses a significant threat to humankind (2). One emerging multidrug-resistant fungus, *Aspergillus fumigatus*, is a critical human fungal pathogen and the causative agent of invasive and chronic aspergillosis (3, 4). Azoles, which target the Cyp51 enzyme (also called Erg11 in yeast) in the ergosterol biosynthesis pathway, are the current frontline therapy against most filamentous fungal infections (5). Azole efficacy *in vivo* is increasingly compromised by acquired resistance driven by both cyp51A mutations and non-Cyp51 mechanisms (6). However, clinical treatment failures also occur with susceptible isolates as determined by standard laboratory drug susceptibility tests (7), resulting in persistent infections (8). It is thus likely that alternative ill-defined mechanisms enable pathogenic fungi to overcome *in vivo* drug pressures.

One such alternative mechanism is antifungal tolerance. Antifungal tolerance is defined as the ability of a microbial population to survive or grow at drug concentrations higher than the MIC, albeit slowly, without a change in MIC (9, 10). Tolerance mechanisms are far less well understood than resistance mechanisms in human pathogenic fungi, with seminal studies largely focused on the pathogenic yeast (11–14). In bacteria, tolerance may precede resistance emergence by persisting under antibiotic pressure long enough to acquire and fix stable mutations (15–18). It is unclear if tolerance is a prerequisite for azole resistance in *A. fumigatus* and if so, how a strain can transition from tolerance to true resistance. Moreover, regulators of antifungal tolerance in pathogenic filamentous fungi are ill-defined.

Given the critical role chromatin plays in sensing environmental changes and facilitating adaptation through transcriptional rewiring in response to stress (19, 20), we decided to investigate unexplored families of chromatin regulatory proteins in *Aspergillus fumigatus* for their role in azole drug responses. One such family includes the inhibitory growth domain (ING; Pfam domain: PF12998) containing proteins that are conserved in eukaryotes (21). *A. fumigatus* possesses three proteins with an ING domain. Interestingly, one particular family member, AFU3G11940 (*ingB*), had allelic variants emerge in a set of longitudinal isolates collected from a patient with cystic fibrosis that displayed an azole tolerance-like phenotype (22). Moreover, in a series of longitudinally collected isolates from a patient with chronic granulomatous disease with reduced susceptibility to azoles, *ingB* variant alleles were also detected (23). Here, we found that loss of *ingB* results in tolerance to azoles *in vitro* and *in vivo* in a murine model of invasive pulmonary aspergillosis. Surprisingly, we observed that this azole-tolerant mutant rapidly acquires resistance when exposed to azoles *in vitro*. Notably, a frameshift mutation in the putative 20S proteasome gene (*umpA*) arises in *ingB* null mutants and results in reduced growth and conidiation, an increase in vegetative mycelium, and resistance to multiple azoles. Therefore, our study addresses a significant knowledge gap by identifying novel gene(s) and mechanisms related to *A. fumigatus* azole drug tolerance and its role in the emergence of drug resistance.

## RESULTS

### Loss of *ingB* results in tolerance to triazoles

**Fig 1:**
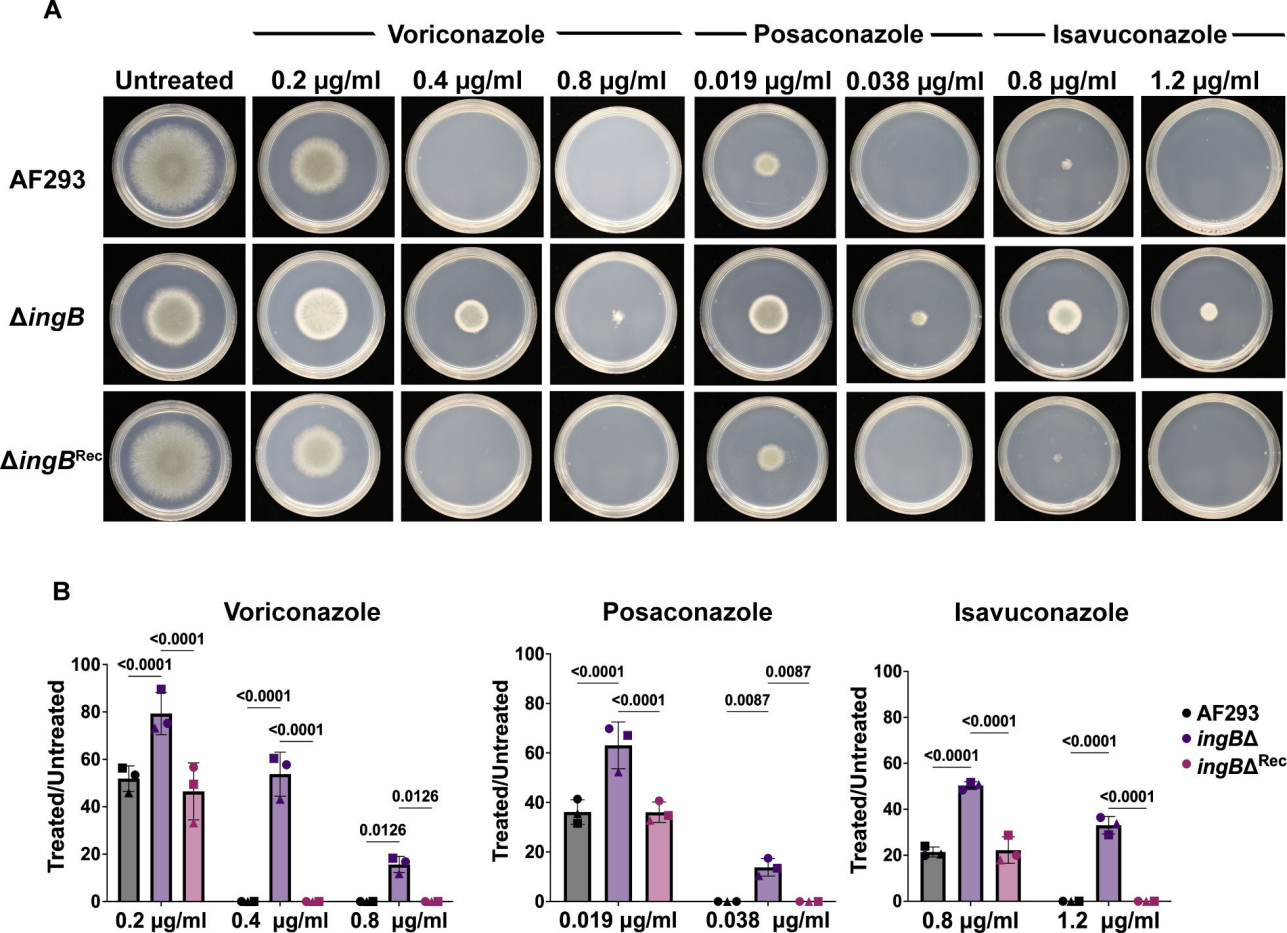
Loss of *ingB* results in tolerance to azoles on solid agar plates. (**A**) A total of 1,000 conidia were inoculated in GMM alone or in the presence of voriconazole, posaconazole, or isavuconazole at 37°C, 5% CO_2_. After 72 h, the radial growth was measured, and the images were taken. (**B**) Two-way analysis of variance (ANOVA) with Dunnett’s multiple comparison test was used to determine significance. The *P*-value is indicated on the graph. Data are representative of three biological replicates (indicated by symbols).

The genetics strategy employed for the generation of Δ*ingB* and Δ*ingB*^Rec^ is detailed in Materials and Methods, and the validation of strains is shown in Fig. S1. Loss of *ingB* resulted in a modest reduction in colony biofilm growth on glucose minimal medium (GMM) and minimal morphological changes that were both reconstituted with re-introduction of the wild-type allele (Fig. 1). We next tested Δ*ingB* susceptibility to azoles using the standardized Clinical and Laboratory Standards Institute (CLSI) microdilution method. Loss of *ingB* did not result in a change in susceptibility to voriconazole, itraconazole, or posaconazole (Table 1). However, while growth in the presence of 0.2 µg/mL voriconazole in agar resulted in about a 50% reduction in colony diameter of the WT and reconstituted strains, and no growth was observed at 0.4 µg/mL or 0.8 µg/mL, which are concentrations above the MIC, the Δ*ingB* strain only showed a ~20% reduction in colony diameter in the presence of 0.2 µg/mL voriconazole, a 50% reduction at 0.4 µg/mL, and a 90% reduction at 0.8 µg/mL voriconazole (Fig. 1). These data suggest that *ingB* is an important mediator of azole susceptibility and its loss results in a drug tolerance phenotype (growth above the MIC established in standardized drug susceptibility assays).

**TABLE 1:** Minimum inhibitory concentrations of strains generated in the study

Strains	Voriconazole	Itraconazole	Posaconazole	Isavuconazole	Amphotericin B
AF293	0.25	0.25	0.031	1	1
Δ*ingB*	0.5	0.5	0.031	2	1
Δ*ingB*^Rec^	0.25	0.25	0.031	1	1
Δ*umpA*	2	>8	0.125	4	2
Δ*umpA*^Rec^	0.25	0.25	0.031	1	1
Δ*umpA*^Ser55fs^	2–4	>8	0.125	4	2
Δ*ingB umpA*^Ser55fs^ (colony 11)	4	>8	0.125	8	2
colony 11 *umpA*^AF293^	0.5	0.5	0.031	2	1

To test whether this tolerance phenotype was specific to voriconazole, we conducted the same assay with other clinically relevant azoles. In the presence of 0.019 µg/mL posaconazole, the WT and reconstituted strains showed approximately a 75% reduction in growth. In contrast, the Δ*ingB* strain showed a 40% reduction in growth (Fig. 1). At the concentration above the MIC, the WT and reconstituted strains showed no growth, while the Δ*ingB* had persistent growth. The MIC for isavuconazole is 1 µg/mL (Table 1). Consistent with observations with the above data, at a sub-MIC of 0.8 µg/mL isavuconazole, the WT and reconstituted strains showed approximately an 80% reduction in growth, whereas Δ*ingB* showed a 50% reduction in growth (Fig. 1). At concentrations slightly above the MIC (1.2 µg/mL), 70% growth reduction was observed in Δ*ingB*, and no growth was observed in WT and the reconstituted strains. Similar observations were made with itraconazole (Fig. S2. Taken together, our data suggest that loss of *ingB* not only results in a strain with less susceptibility to azoles than WT at sub-MICs but also one that can grow at azole concentrations above the MIC on solid medium, despite no change in MIC. We conclude that loss of *ingB* leads to azole tolerance.

### Δ*ingB* biofilms are less susceptible to azoles *in vitro* and *in vivo*

**Fig 2:**
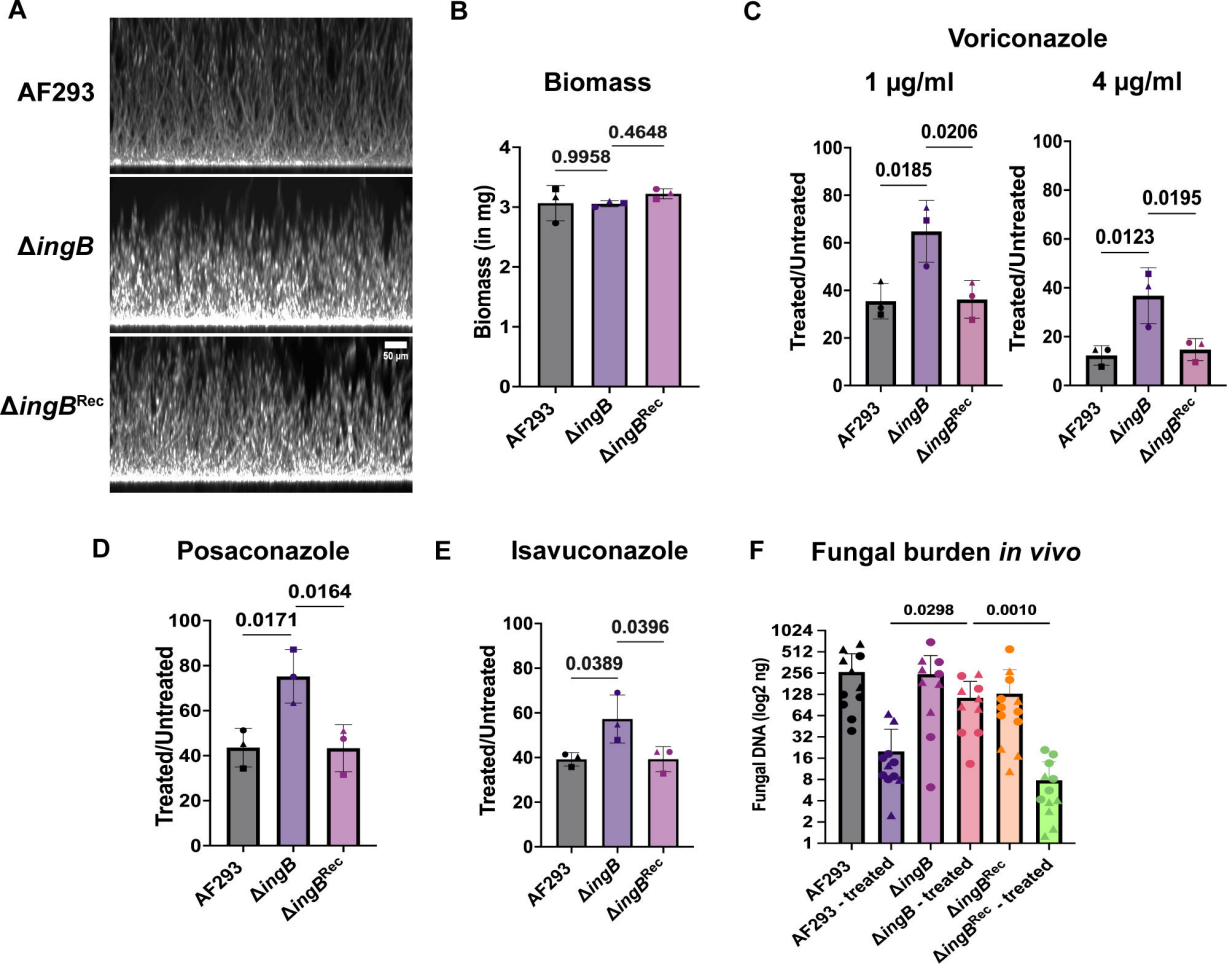
Loss of *ingB* results in reduced biofilm susceptibility to azoles *in vitro* and *in vivo.* (**A**) Representative images of 16 h biofilms stained with calcofluor white. The scale bar is indicated on the image. (**B**) No difference in biomass was observed among the groups at 16 h, before drug treatment. For each technical replicate, biomass from two wells of a six-well plate was pooled. One-way analysis of variance (ANOVA) was not significant (*P* = 0.4912). The experiment was repeated three times independently (indicated by symbols) with three technical replicates in each experiment. Dunnett’s multiple comparison test was used to compare each group with the mutant, and the *P*-value is indicated in the graph. For drug susceptibility assays, 16 h biofilms grown at 37°C, 5% CO_2_ were treated with (**C**) 1 or 4 µg/mL voriconazole, (**D**) 0.2 µg/mL posaconazole, and (**E**) 2 µg/mL isavuconazole for 4.5 h. The medium was then removed and replaced with fresh minimal growth medium. After 16 h, biomass was collected. Treated-to-untreated biomass ratios are shown in the graphs for each concentration. The experiment was repeated three times independently (indicated by symbols). One-way ANOVA was significant for voriconazole at 1 µg/mL (*P* = 0.0166) and 4 µg/mL (*P* = 0.0128), posaconazole (*P* = 0.0141), and isavuconazole (P = 0.0345). Dunnett’s multiple comparison test was used to compare each group with the mutant, and the *P*-value is indicated in the graph. (**F**) Loss of *ingB* results in reduced susceptibility to voriconazole *in vivo*. Four- to six-week-old CD-1 mice were immunosuppressed, infected, and treated with voriconazole. Fungal burden was assessed through quantitative PCR (qPCR) quantitation of *A. fumigatus* 18S rDNA. The experiment was repeated two times, as indicated by symbols. The Kruskal-Wallis test was significant (*P* < 0.0001). Dunn’s multiple comparison test was used to compare treated groups, and the *P*-value is indicated on the graph.

Previous work has shown that biofilm growth of filamentous fungi significantly reduces antifungal drug susceptibility (24, 25). We wondered if strain displaying a tolerance phenotype with agar surface colony biofilms would also possess altered drug susceptibility in a submerged biofilm culture model. We first compared the biofilms of Δ*ingB* with those of WT to ensure that biofilm formation and growth in this model are similar. As presented in Fig. 2A, Δ*ingB* biofilm morphology is similar to the WT (AF293) and the reconstituted strain at 16 h of development when some wild-type drug susceptibility still exists. The biofilm morphology is characterized by adherence to the base of the substrate and hyphal growth along the XY and Z-axis over time. Additionally, no significant differences in biofilm total biomass were observed between WT, Δ*ingB*, and the reconstituted strain (Fig. 2B). Therefore, we next tested biofilm antifungal susceptibility in the presence and absence of *ingB*.

As presented in Fig. 2C, treatment with 1 µg/mL voriconazole (4× MIC) resulted in a ~65% reduction in biofilm growth at the tested time point in the WT and the reconstituted strain, whereas a significant reduction in susceptibility was observed in Δ*ingB* biofilms (~40% decrease in biomass growth, compared to untreated). At the large 4 µg/mL dose of voriconazole, compared to untreated controls, the WT and the reconstituted strain had a ~90% growth reduction, while Δ*ingB* had a ~65% reduction. Similar results were observed with posaconazole (Fig. 2D). The WT and the reconstituted groups had a ~60% reduction in biomass growth when compared to untreated controls. However, Δ*ingB* only had a 30% reduction. The Δ*ingB* biofilms are also less susceptible to isavuconazole (Fig. 2E). Taken together, Δ*ingB* tolerance to azoles is not dependent on its morphological form, as both Δ*ingB* conidia/colony biofilms and developing submerged biofilms show reduced susceptibility to azoles in separate, distinct assays. As biofilm growth is relevant *in vivo*, we wondered if the Δ*ingB* azole tolerance would be relevant in a pre-clinical IPA (Invasive Pulmonary Aspergillosis) animal model. Therefore, we next tested whether loss of *ingB* impacted fungal burden in a murine model of invasive pulmonary aspergillosis with and without voriconazole treatment.

The fungal burden between the groups not treated with voriconazole across strains was similar, suggesting that *ingB* is dispensable for fungal fitness and disease progression in this murine IPA model (Fig. 2F). Loss of *ingB* also did not alter murine survival outcomes in the triamcinolone model of IPA (Fig. S3). However, mice challenged with the WT and the reconstituted strains and treated with voriconazole resulted in a ~16-fold decrease in pulmonary fungal burden. In contrast, mice challenged with Δ*ingB* and treated with voriconazole only resulted in a 2.5-fold decrease in fungal burden (Fig. 2F). Consistently, Δ*ingB*-treated groups show statistically significantly higher fungal burden than the WT or reconstituted group treated with voriconazole (Fig. 2F). Therefore, consistent with the observations made *in vitro* in two distinct assays, loss of *ingB* favors survival and growth under voriconazole treatment *in vivo*.

### Loss of *ingB* leads to repression of genes involved in ergosterol biosynthesis and an increase in iron starvation response

We next explored the potential mechanism behind the IngB-mediated azole tolerance phenotype. A previous study identified IngB as part of the Nua3 Histone Acetyltransferase complex, which catalyzes the acetylation of H3K9 and H3K14 (26). IngB orthologs are also found to be part of the same complex in other fungal species (27–29). Given its role in gene regulation and expression, we utilized RNA-seq to understand the differences in gene expression between Δ*ingB* and WT. The loss of *ingB* resulted in a significant change in the transcript abundance of 1,225 genes (*P* < 0.05), approximately 12% of the annotated genes in the *A. fumigatus* genome. The predominant number of transcripts in the Δ*ingB* mutant are reduced compared to the WT, suggesting that IngB likely functions primarily as a positive regulator of gene expression. The list of all gene CPM values and fold change expression values is detailed in Tables S1 to S3.

**Fig 3:**
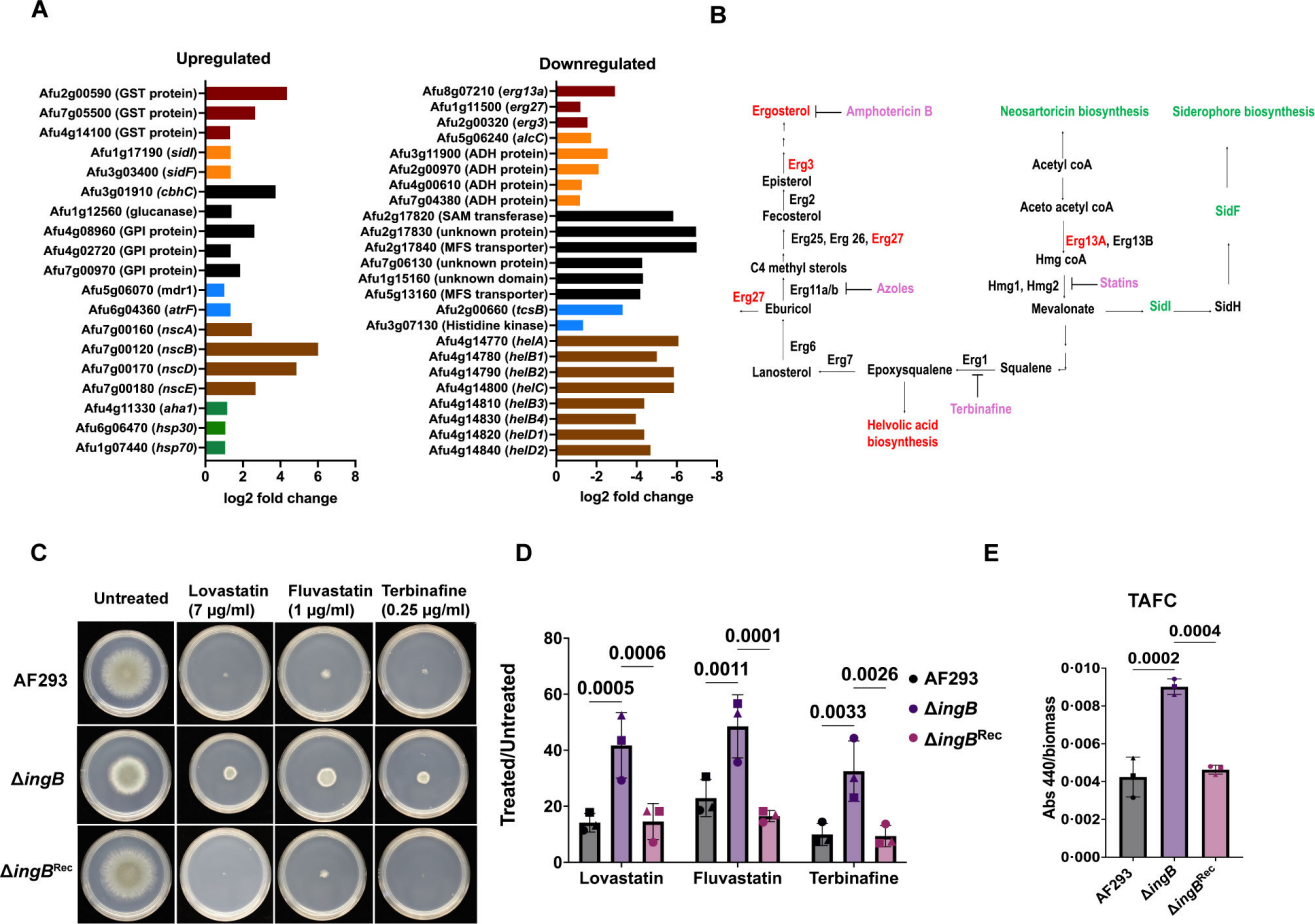
Deletion of *ingB* results in the repression of genes in ergosterol biosynthesis and alters global cellular stress response signature. (**A**) Comparison of Differentially Expressed Genes (DEG) in Δ*ingB* vs WT. RNA-seq was performed on 16 h biofilms of AF293 and Δ*ingB*. There were 1,225 DEG between Δ*ingB* and AF293. Of these, the expression of 536 genes was significantly changed by at least twofold, and their CPM values were more than 10. Select upregulated and downregulated genes (twofold difference, *P* < 0.05, CPM > 10) in the mutant are shown in the figure: Upregulated—genes involved in putative glutathione S-transferases (dark red), siderophore biosynthesis (orange), cell wall (black), drug transporters (blue), neosartoricin biosynthesis (brown), and heat-shock protein genes (green). Downregulated—genes involved in ergosterol biosynthesis (dark red), alcohol dehydrogenases (orange), membrane integrity (black), two-component system (blue), and helvolic acid biosynthesis (brown). (**B**) The downregulation (indicated in red) of *erg13a*, *erg3*, and *erg27*, alongside an increase (indicated in green) in siderophore biosynthesis genes and neosartoricin biosynthesis was observed. (**C**) The conidia of each strain were inoculated on plates containing no drug, lovastatin (7 µg/mL) , fluvastatin (1 µg/mL), and terbinafine (0.25 µg/mL). Radial growth was measured, and plate images were taken. (**D**) Treated-to-untreated ratios are shown. Two-way analysis of variance (ANOVA) with Dunnett’s multiple comparison test was used to compare each group with the mutant, and the *P*-value is indicated in the graph. The data shown are representative of three bioreps (indicated by symbols). (**E**) Each strain was cultured in flasks containing iron-free GMM. The flasks were incubated at 37°C, 5% CO_2_, and 200 rpm. TAFC production per weight of biomass was calculated. The data shown are representative of three bioreps (indicated by symbols). One-way ANOVA was significant (*P* = 0.0002). Dunnett’s multiple comparison test was used to compare the mutant with the WT and the reconstituted groups. The *P*-value is indicated on the graphs.

Several key genes involved in *A. fumigatus* growth, metabolism, stress response, and secondary metabolism, whose transcripts were significantly altered in the absence of *ingB*, are highlighted in Fig. 3A. We focused on pathways and mechanisms known for a role in pathogenesis and drug susceptibility. Among the genes with increased transcript levels were several members of the heat shock protein family, which play a crucial role in mitigating stress and enhancing antifungal resistance (30). Several transcripts of genes encoding putative glutathione S-transferases, which are generally involved in cellular detoxification and protection against oxidative stress (31), were also increased in the Δ*ingB* mutant (Fig. 3A). We also observed a modest increase in transcripts of *mdr1* (32) and *atrF* (33) drug transporters (Fig. 3A).

Among the genes with decreased transcript levels in the absence of *ingB* are several putative genes encoding proteins involved in maintaining cell membrane integrity (Fig. 3A). Transcripts of two-component signal transduction systems, which perceive extracellular signals and activate downstream response pathways, were also significantly reduced in the Δ*ingB* mutant (34). Genes encoding transcripts related to primary and secondary metabolism were altered, including those involved in the mevalonate pathway, several alcohol dehydrogenases, and the helvolic acid pathway (Fig. 3B).

As acetyl-CoA is a key building block for fungal sterols (35), we were particularly intrigued by transcripts related to acetyl-CoA metabolism with loss of *ingB*. Acetyl-CoA acts as a precursor to several primary and secondary metabolism pathways in addition to its key role in sterol and lipid synthesis. As shown in Fig. 3B, acetyl-CoA acts as a precursor to the mevalonate pathway (35) and polyketide synthesis (36). Transcripts of the genes involved in neosartoricin biosynthesis were increased in the absence of *ingB*; transcripts of the early mevalonate pathway gene *erg13A*, encoding a hydroxymethylglutaryl-CoA (HMG-CoA) synthase, were significantly decreased. Mevalonate serves as a precursor to the ergosterol pathway, or it can be directed toward the synthesis of siderophores (37). Interestingly, transcripts of genes involved in siderophore biosynthesis, *sidI* and *sidF*, were significantly increased with *ingB* loss by 2.71-fold (Fig. 3A). With regard to helvolic acid synthesis, squalene, also a sterol precursor, is converted to (3S)−2,3-oxidosqualene that can be incorporated into the ergosterol biosynthesis pathway or utilized for helvolic acid biosynthesis (38). Perhaps correspondingly, transcripts of all genes involved in helvolic acid biosynthesis and transcripts of several genes in the ergosterol pathway were decreased (Fig. 3B). Together, the data suggest that IngB is a global regulator of primary and secondary metabolism, stress responses, and cell membrane homeostasis in *A. fumigatus*. A chromosome map showing the affected clusters of genes with *ingB* loss is in Fig. S6.

### Complex metabolism and stress response regulation likely drive Δ*ingB* tolerance to azoles

We hypothesized that loss of *ingB* results in global transcriptional rewiring leading to a constitutively active stress response signal, leading in part to a critical metabolic trade-off by shunting mevalonate pathway precursors away from ergosterol biosynthesis toward siderophore production. We reasoned that this global re-wiring may yield tolerance or resistance to other sterol-targeting antifungals. Perhaps consistent with this hypothesis, treating WT and the reconstituted strain with 7 µg/mL lovastatin, which targets HMG-CoA reductase, resulted in approximately 85%–90% reduction in growth of the WT, whereas Δ*ingB* showed only a 60% growth decrease (Fig. 3C and D). Similar observations were also made with fluvastatin, which also targets HMG-CoA reductase, where Δ*ingB* is statistically significantly less susceptible than the WT and the reconstituted strain (Fig. 3C and D). Additionally, treatment with terbinafine, which targets the Erg1 squalene epoxidase, resulted in about 90% reduction in colony size in the WT and the reconstituted strain, but a 70% reduction in Δ*ingB* colony size (Fig. 3C and D). Intriguingly, susceptibility to amphotericin B was not altered in Δ*ingB* (Fig. S4). However, consistent with the observed increase in siderophore biosynthesis transcripts, TAFC siderophore production per gram of fungal biomass was increased in Δ*ingB* compared to WT and the reconstituted strain (Fig. 3E). Taken together, these data support the transcriptomics analysis and indicate that loss of *ingB* likely perturbs the flux of key metabolites and the generation of toxic intermediates involved in the production of ergosterol.

### Δ*ingB*-mediated tolerance drives the selection of acquired drug resistance

**Fig 4:**
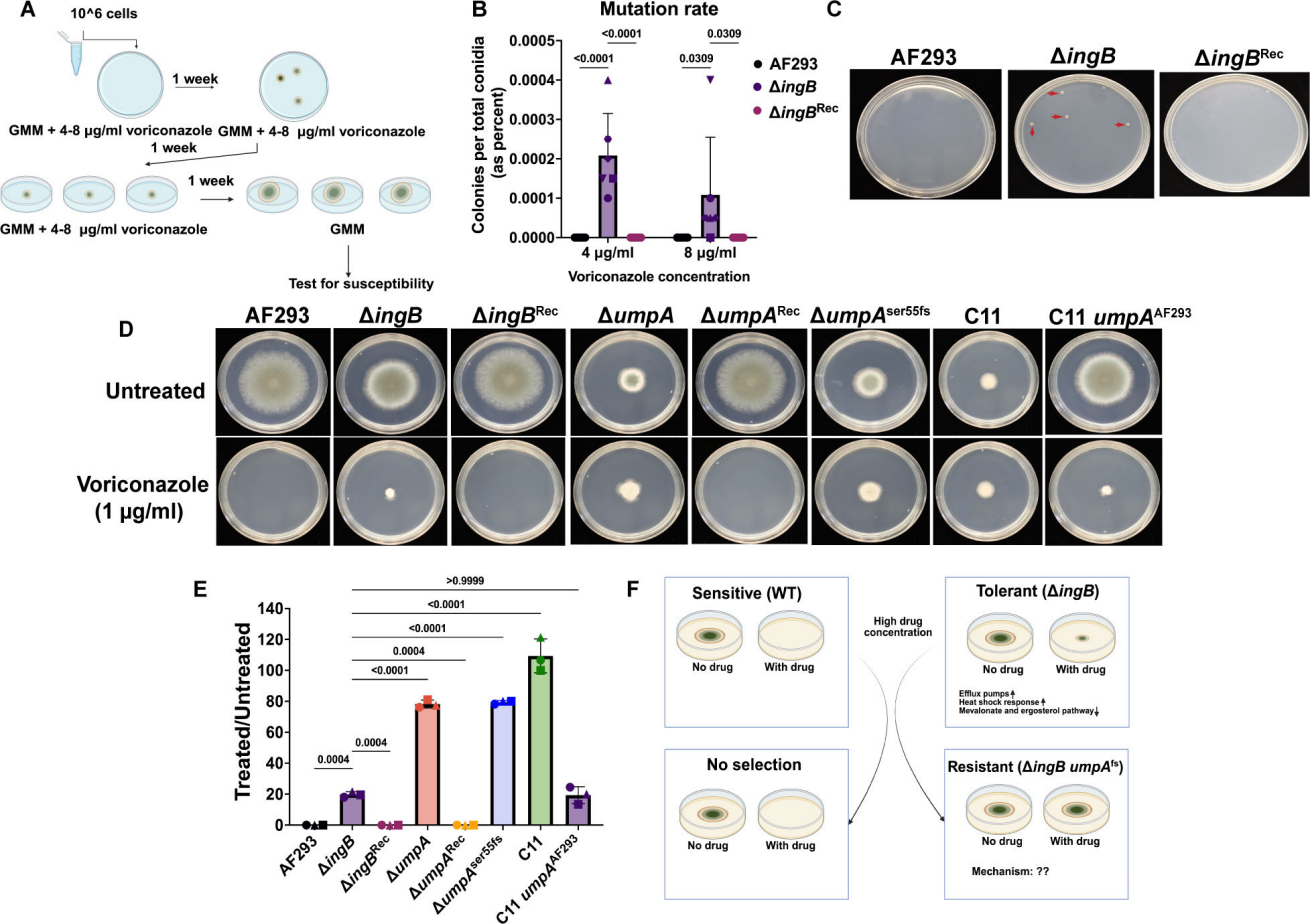
Loss of *ingB* results in the selection of azole-resistant strains with a frameshift mutation in *umpA*. (**A**) The experiment design for the selection of azole-resistant strains. (**B**) The mutation rate was calculated by the total number of colonies per total population of conidia at two different voriconazole concentrations. Each point represents a different biorep. Two-way analysis of variance (ANOVA) for the column factor (strain comparison) was significant (*P* < 0.0001). For the row factor (different concentrations), no statistical significance was observed (*P* = 0.1865). Dunnett’s multiple comparison test was used to compare groups at both concentrations, and the *P*-value is indicated on the graph. (**C**) Representative plate images from the selection experiment are shown. The red arrows indicate colonies on the plate. (**D**) *umpA^Ser55fs^* drives azole resistance. The conidia of the strains were spotted on GMM containing 0 or 1 µg/mL voriconazole for 72 h, and radial growth was measured. (**E**) Quantification of treated-to-untreated ratios of plates shown in panel D. One-way ANOVA was significant (*P* < 0.0001). Dunnett’s multiple comparison was used to compare each group with Δ*ingB*, and the *P*-value is indicated on the graph. Each biological replicate is indicated by a symbol in the graph. (**F**) Azole tolerance accelerates the selection of acquired resistance. Drugs such as azoles inhibit the growth of a sensitive *A. fumigatus* strain (WT). However, a tolerant strain (∆*ingB*) is less susceptible to azoles, despite no change in its MIC. This tolerance response is characterized by modest upregulation of stress responses such as heat shock proteins and efflux pumps, and downregulation of genes involved in the mevalonate and ergosterol pathways. When both the sensitive and the tolerant strains are exposed to a high concentration of azoles, this results in the killing of all sensitive cells. However, a sub-population of the tolerant population can gain mutations to become resistant, thereby providing a selection advantage. The model figure was created in BioRender. Cramer, R. (2026) https://BioRender.com/d1tbpn4.

As a putative loss-of-function *ingB* allele was discovered in a series of azole-resistant isolates from a person with chronic granulomatous disease (23), and loss of *ingB* in our studies promotes azole tolerance, likely through modulation of fungal metabolism associated with sterol production, we asked whether the tolerant *ingB* strain would be prone to development of true antifungal resistance. To address this significant knowledge gap, we conducted a selection experiment where we inoculated the parent AF293 (sensitive), Δ*ingB* (tolerant), and reconstituted strain (sensitive) at voriconazole concentrations 16×–32× above the MIC (Fig. 4A).

As presented in Fig. 4B and C, despite a high inoculation concentration of conidia, we did not recover any colonies from the sensitive strains over six biological replicates, while dysmorphic but growing colonies were recovered from Δ*ingB* inoculated plates. The approximate mutation rate for *ingB* under this selection was calculated by the number of colonies that emerged per total conidia inoculated in each biological replicate (39). A mutation rate in Δ*ingB* of 0.002% and 0.001% was observed at 4 and 8 µg/mL of voriconazole, respectively. Colonies obtained on the Δ*ingB* drug plates were compact and appeared reddish. When these colonies were patched onto GMM with no drug, most colonies were compact and had increased vegetative mycelium (Fig. S5A). All the colonies that were tested were resistant to voriconazole (Fig. S5B). Therefore, our data suggest that tolerance conferred by the absence of IngB promotes the emergence of antifungal-resistant mutants upon high-concentration azole exposure.

### *umpA^ser55fs^* is a novel mutation that confers triazole resistance

To determine how the tolerant strain (Δ*ingB*) became resistant, we utilized whole-genome sequencing on five independent colonies that exhibited azole resistance following voriconazole exposure (Fig. S5). Colony 1 had a frameshift mutation in Afu5g08450 (UBA_TAP-C-like protein), colony 9 had a frameshift mutation in Afu5g06790 (NTF-2 domain protein), and colony 11 had a frameshift mutation in Afu5g10740, which is a conserved ortholog of the proteasome maturation factor *umpA* in fungi. We decided to investigate further if the *umpA^ser55fs^* allele conferred azole resistance. We generated a Δ*umpA* mutant in the WT background and then reconstituted the strain with the WT *umpA* allele or with the colony 11 allele (which contains the *umpA^ser55fs^* allele; see Materials and Methods). Alternatively, we also reconstituted colony 11 with the WT *umpA* allele from AF293. Strain validation data are shown in Fig. S1.

If the frameshift mutation results in a loss of function, we would expect the null mutant to exhibit the same phenotype as the evolved allele. Conversely, restoring the wild-type *umpA* allele in colony 11 should restore azole susceptibility. Consistent with our hypothesis, both *umpA^ser55fs^* and Δ*umpA* strains are resistant to voriconazole, itraconazole, posaconazole, and isavuconazole (Table 1). We did not observe a drastic change in MIC with amphotericin B. As expected, restoring the WT *umpA* allele in colony 11 restored antifungal susceptibility to Δ*ingB* levels (Table 1).

We also utilized a second plate-based agar assay to test for azole susceptibility (Fig. 4D and E). As expected, we did not observe any growth of the WT and reconstituted strains on plates containing 1 µg/mL voriconazole. Interestingly, although colony 11, *umpA^ser55fs^*, and the Δ*umpA* strains have the same MIC, colony 11 exhibits more growth in the presence of high concentrations of azoles. As expected, restoring the WT copy of *umpA* in colony 11 also restored the tolerance to Δ*ingB levels*. Taken together, these data identify a new antifungal resistance factor, *umpA*, in pathogenic filamentous fungi and suggest that tolerance may lead to the emergence of azole drug resistance.

## DISCUSSION

Studies from bacterial species have recognized antibiotic tolerance as a significant driver of treatment failures, particularly in chronic infections (40). Antibiotic tolerance also serves as a stepping stone to the development of acquired antibacterial resistance (15, 16). Despite recent studies focused on identifying the genetic drivers of azole tolerance in fungi (41, 42), a significant gap remains in understanding how *A. fumigatus* survives or even grows under high azole concentrations and how this survival may impact treatment outcomes. Addressing these questions is expected to aid in determining the clinical significance of azole tolerance and whether tolerance may contribute to the development of clinical resistance in the setting of chronic aspergillosis. If so, identified mechanisms may assist in the development of novel antifungal strategies to mitigate persistent infections in chronic aspergillosis before true resistance emerges.

Here, we identified IngB as a novel regulator of filamentous fungus azole susceptibility whose loss leads to an azole tolerance phenotype and true azole resistance under azole selective pressure. The azole tolerance in Δ*ingB* is not dependent on morphology or developmental stage as it was observed in two distinct assays. Importantly, the Δ*ingB in vitro* azole tolerance phenotype translated *in vivo* to a murine model of invasive pulmonary aspergillosis as an increase in Δ*ingB* fungal burden upon voriconazole treatment compared to the control strains was observed. These data at least raise the possibility that azole-tolerant strains in human infections may be more difficult to eradicate. Support for this possibility comes from the emergence of a putative loss-of-function *ingB* in *A. fumigatus* isolates longitudinally obtained from a patient with chronic granulomatous disease that ultimately became azole resistant over time (23). However, a limitation of the current study in supporting this possibility further is the tolerance strain studied is based on a loss of IngB. Further studies are needed to determine the role of IngB and other other tolerance-promoting mutations in long-term chronic infection fungal persistence and treatment failures.

An outstanding question is how loss of *ingB* promotes azole tolerance. Loss of *ingB* results in chromatin-dependent transcriptional remodeling (Fig. S6) affecting the ergosterol pathway, iron starvation, secondary metabolism, oxidative metabolism, cell wall homeostasis, glutathione S-transferases, drug transporters, and heat shock proteins such as Hsp90 that impact antifungal drug efficacy (30, 32, 33, 43, 44) (Fig. 3A). Consequently, the altered physiological state resulting from global transcriptome re-wiring upon IngB loss likely reduces cellular dependence on ergosterol biosynthetic flux and increases tolerance to azoles and other pathway inhibitors. In fact, the slower growth of the *ingB* null mutant on agar surfaces likely reflects these altered metabolic fluxes that likely would alter (lower) the generation of toxic metabolites produced by azole and terbinafine treatments. With a slower metabolism that leads to slower growth, it is likely that the demand for sterol turnover also decreases, perhaps explaining the lack of change in ergosterol targeting amphotericin B susceptibility with loss of IngB. Previously, in *A. fumigatus*, a critical metabolic link between the ergosterol pathway and the siderophore biosynthesis pathway was investigated (37). Under iron starvation, mevalonate is diverted toward siderophore biosynthesis, which results in reduced metabolites for the ergosterol pathway. Based on this and the transcriptome data of the *ingB* mutant strain, we hypothesized that loss of *ingB* results in metabolic rewiring that reduces flux through the ergosterol biosynthesis pathway. Consistent with our hypothesis, Δ*ingB* showed an increase in TAFC production. Surprisingly, the expression levels of transcription factors HapX, SreA, or SrbA, known regulators of iron starvation and the ergosterol pathway (45–47), were not significantly different in *ΔingB*. Additional studies are warranted to further understand mechanisms of IngB gene regulation.

The phenomena of azole tolerance and resistance have largely been studied separately; the relationship between them is a significant knowledge gap in *A. fumigatus*. In *Candida albicans*, mechanisms of drug tolerance and resistance are parallel mechanisms and occur at different drug concentrations, and a transition from tolerance to resistance does not appear to happen (14). Azoles are fungicidal at high concentrations against *A. fumigatus*, but largely fungistatic against *C. albicans*. It is possible that fungistatic drugs do not generate the same selection pressure as fungicidal drugs at high drug concentrations. With *A. fumigatus*, our data suggest that a single high concentration azole exposure of a tolerant strain due to loss of IngB, but not a sensitive strain, is sufficient to develop azole resistance in *A. fumigatus* (Fig. 4). Hence, tolerance is not merely a survival mechanism, but also plays an active role in shaping antifungal resistance, at least in the absence of IngB. It will be important to test non-IngB-dependent tolerant strains to see if azole resistance also arises in those ill-defined genotypes. Specific metabolic perturbations that occur in the absence of *ingB* may lead to the promotion of azole resistance that could be absent in other tolerant strains. These future studies will inform not only mechanisms of tolerance but also a further understanding of the biochemical pathways mediating azole resistance. As echinocandins such as micafungin are fungistatic against *A. fumigatus*, it would be interesting to test whether a link between tolerance and resistance also exists here. Alternatively, passaging *A. fumigatus* at sub-MICs of azoles may also reveal the necessity of tolerance in the evolution of resistance.

An important observation that arose in these studies is the identification of a novel frameshift mutation in Afu5g10740 (*umpA*) that leads to azole resistance. UmpA is highly conserved in fungi but has not been studied to date in *A. fumigatus*. In *Saccharomyces cerevisiae*, it is required for the correct maturation of the 20S proteasome for non-lysosomal protein degradation (48). Given the nature of the mutation, we hypothesized it to be a loss-of-function mutation, and indeed, both *umpA^ser55fs^* and the Δ*umpA* strains are resistant to azoles. On solid agar plates at 4× MIC, *umpA^ser55fs^* and Δ*umpA* show a reduction in colony size by about 40%; however, Δ*ingB umpA^ser55fs^* (colony 11) shows no reduction at 4×, suggesting that epistatic interactions occur in Colony 11 under azole stress.

Rpn4 is a transcription factor in *C. albicans* that activates proteasome genes to overcome the proteotoxicity induced by fluconazole (49). Loss of *rpn4* results in a loss of azole tolerance. Even in *A. fumigatus*, exposure to azoles causes oxidative and proteotoxic stress (50), and the damaged proteins are likely to be removed through ubiquitin-mediated degradation. This raises the question: How is Δ*umpA* or *umpA^ser55fs^* resistant to azoles? In *Saccharomyces cerevisiae*, the proteasome is also involved in maintaining genomic stability (51). Loss of *ump1* results in a hypermutator phenotype, characterized by an increased frequency of spontaneous mutations (52, 53). This is because proteasome activity is reduced in the Δ*ump1* strain, which prevents the rapid degradation of mutagenic DNA repair factors, specifically components of the translesion synthesis pathway such as polymerase Pol 𝜁. In *A. fumigatus*, hypermutator strains resulting from impaired DNA mismatch repair increase the frequency of azole resistance (39, 54). Hence, it is possible that *umpA^ser55fs^* mutation and Δ*umpA* could also result in a hypermutator phenotype that could further drive antifungal resistance. Slower degradation of the drug target Cyp51A/B or hyperactivation of a stress response pathway in the presence of azoles could also drive resistance in Δ*umpA*. Future studies are needed, though the current study suggests allelic variation in *umpA* and related proteasome components should be investigated in clinical isolates with unexplained azole susceptibility phenotypes.

In conclusion, we identified IngB as a regulator of antifungal azole susceptibility whose loss results in a drug tolerance phenotype both *in vitro* and *in vivo* in a murine model of IPA. The ingB null mutant is characterized by increased tolerance, accompanied by reduced expression of genes in the ergosterol pathway, increased expression of genes involved in drug efflux, iron starvation response, and a general cell stress response, including heat shock proteins (Fig. 4F). Our study further revealed that loss of IngB accelerated the selection of acquired resistance in part through mutation of the putative 20S proteosome maturation factor, UmpA (Fig. 4F). Our future work aims to determine how UmpA drives azole resistance. Thus, our study not only characterized a novel regulator of the azole drug response in *A. fumigatus*, but also identified a link between azole tolerance and resistance mechanisms. Pending additional studies, future antifungal therapies designed to reduce drug tolerance could be an effective strategy to reduce the emergence of antifungal resistance when long-term therapies are required.

## MATERIALS AND METHODS

### Strains and culture

The reference strain AF293 (55) was used as the background strain to generate mutants. The list of strains used in the study is provided in the supplementary data. The cryostocks were maintained in 25% glycerol at −80℃. Three days prior to the experiment, the strains were streaked on GMM agar with trace elements and salt solution (56), and the plates were incubated at 37 °C, 5% CO_2_. The conidia were collected in 0.01% tween-80 water and filtered through autoclaved Miracloth. All *in vitro* experiments in the study were performed in liquid or solid GMM at 37°C, 5% CO_2_.

### Generation of *Aspergillus fumigatus* strains

Both *ingB* and *umpA* null mutants were generated using CRISPR-Cas9 technology as previously described (57). Two gRNA targeting sequences, one upstream and one downstream of the gene, were designed. For transformation, AF293 protoplasts (58) were transformed with a preformed Cas9-RNP complex (containing Cas9 and gRNA) and 2 μg of a repair construct. The repair construct consisted of a hygromycin expression cassette with 35 bp overhang sequences adjacent to the Cas9-targeted sites. The transformants were selected on GMM with 1.2 M sorbitol (SMM) and hygromycin (175 μg/mL). The mutants were confirmed by junction PCRs and by checking for the absence of the open reading frame in the mutant. RT-qPCR was also used to confirm the expression in the null mutants and the reconstituted strains described below.

For the generation of reconstituted strains, the *ingB* or *umpA* gene locus (sequences obtained from FungiDB) was amplified from the AF293 genome. The resulting product was integrated into a plasmid containing pyrathimaine (*ptrA*) selection marker using HiFi (NEB), such that the selection marker lies downstream to the gene locus. For the generation of the allele swap strain, the *umpA^Ser55fs^* gene locus was amplified from colony 11, and the plasmid was generated similarly. The constructs were amplified from the plasmids using PCR and were integrated at the *aft4* safe haven site (59). The integration at the locus was confirmed using junction PCR and checking for the presence of open reading frame. The list of primers and gRNAs used in the study is described in Table S4.

### Azole susceptibility experiments

For the solid plate assay, 1,000 conidia were spotted in the middle of the plate containing various concentrations of azoles. After 72 h, the radial growth was measured, and the plate pictures were taken.

Minimum inhibitory concentration(s) were determined in accordance with the guidelines established in the CLSI M38 document (60), with the exception of using 5% CO_2_ to mimic host conditions.

To test biofilm susceptibility, 10^5^ conidia/mL were cultured in a six-well plate for 16 h and then exposed to azoles for 4.5 h. The drug was removed, and the wells were washed with media and were grown for another 16 h. The biofilms were scraped, collected, washed, lyophilized, and weighed before and after treatments.

### Microscopy

A total of 10^5^ conidia/mL of each strain were cultured in ibidi eight-well glass-bottom slides for 16 h. The biofilms were stained with 25 µg/mL calcofluor white for 15 min. The images were taken at 20× using an Andor W1Spinning Disk Confocal microscope set up on a Nikon Eclipse Ti inverted microscope and were processed using Fiji.

### Murine experiments

The animal protocol was approved by the Institutional Animal Care and Use Committee (federal-wide assurance number A3259-01) at Dartmouth College. The experiments were conducted with strict adherence to the recommendations outlined in the Guide for the Care and Use of Laboratory Animals and with Dartmouth IACUC approval under protocol 00002167 (61).

A total of 20–24 g outbred female CD-1 mice (Charles River Laboratory, Raleigh, NC, USA) were housed in autoclaved cages (3-4 per cage) with HEPA-filtered air, food, and autoclaved water available *ad libitum*. The mice were given 50% grapefruit juice (62–64) 5 days before inoculation, instead of drinking water in their bottles. To induce leukopenia, the mice were intraperitoneally injected with 150 mg/kg cyclophosphamide on 2 days before and 3 days after inoculation. The mice were also subcutaneously administered with 40 mg/kg Kenalog-10 (triamcinolone acetonide; Bristol-Myer Squibb, Princeton, NJ) a day before inoculation. On the day of inoculation, conidia were collected, counted, and resuspended in PBS, and 10^6^ conidia in 40 µL sterile PBS were administered intranasally. The mock group received sterile PBS. After 16 h, the groups receiving voriconazole were given 40 mg/kg in PBS by oral gavage and once daily thereafter for 3 more days. At the end of the experiment (day 4), the mice were sacrificed, and their lungs were harvested, flash-frozen, and lyophilized. The relative fungal burden was assessed through quantitative PCR quantitation of *A. fumigatus* 18S rDNA, as previously described (65).

### RNA-seq transcriptome analysis

For extracting RNA, 10^5^ conidia/mL were inoculated in petri dishes. After 16 h, the biomass was collected in TRI reagent (Invitrogen) and bead-beaten for 1 min. A total of 0.2 mL of chloroform per milliliter of TRI reagent was added to each tube. After a brief incubation, the tubes were centrifuged for 15 min at top speed. The aqueous phase was collected and processed according to the manufacturer’s instructions (RNeasy, Qiagen). After DNase treatment, the quality of RNA was determined by gel electrophoresis, nanodrop, and Qubit. The final samples were submitted to the genomics core at Dartmouth College, which performed fragment analysis and library preparation for PolyA RNA-seq.

The resulting raw reads quality was checked using Initial QC. The raw reads were trimmed for adapters using CutAdapt (66). The reads (~10 million per sample) were then aligned to the AF293 genome obtained from FungiDB, v68 (67) using the STAR package (68). On average, 90% of the reads aligned to the genome. The counts for each gene were compiled using HTSeq software (69). The custom R scripts used for exploratory analysis, differential expression, and figure generation are derived from the pipeline previously described (70). Briefly, the counts file and metadata were used for the exploratory analysis using the DESeq2 package (71). A variance-stabilizing transformation was applied (72), which normalizes the count data and transforms it onto a log2 scale, making the variance largely independent of the mean and thus suitable for PCA plots. The data were then normalized using the trimmed mean of *M*-values method (edgeR) (73), and the edgeR package (74) and limma-voom pipeline (75) were used to identify statistically differentially expressed genes between the mutant and the WT. RNA-seq data sets are available in the Gene Expression Omnibus under the accession number GSE314107 (https://www.ncbi.nlm.nih.gov/geo/query/acc.cgi?acc=GSE314107).

### Siderophore quantification

A total of 5 × 10^7^ conidia were inoculated in baffled flasks containing 100 mL GMM (no iron in trace elements). The flasks were incubated at 37℃, 5% CO_2_, and 200 rpm. After 48 h, the biomass was collected, lyophilized, and weighed. The supernatants were filtered through a 0.45-micron filter, and the pH was measured to ensure it was close to 6.5. To 95 μL of supernatant, 5 μL containing 30 mM FeSO_4_.7H_2_O in a 5 mM HCl solution was added. To control, only a 5 mM HCl solution was added. After 30 min, the absorbance was measured at 440 nm (where TAFC production is detected maximally). The media-only controls (with and without iron) were used to ensure the change of color to orange was observed in fungal samples and not due to the background. The absorbance for each sample with 30 mM iron was subtracted from the sample with solvent only. The result was divided by their respective biomass.

### Whole-genome sequencing

All strains used for the whole-genome sequencing were prepared by culturing 10^5^ conidia/mL in petri dishes. After 24 h, the biomass was collected, frozen, and lyophilized. The resulting biomass was subjected to a bead beater for 1 min, and DNA was extracted using standard protocols. The samples were submitted to Seqcoast (Portsmouth, NH) for short-read Illumina sequencing and variant calling. Samples were prepared for whole-genome sequencing using the Illumina DNA Prep tagmentation kit (#20060059) with Illumina Unique Dual Indexes. Sequencing was performed on the Illumina NextSeq 2000 platform using a 300-cycle flow cell kit to produce 2 × 150 bp paired reads. A total of 1%–2% PhiX control was spiked into the run to support optimal base calling. Read demultiplexing, read trimming, and running analytics were performed using DRAGEN v4.2.7, an on-board analysis software on the Illumina NextSeq 2000. The whole-genome sequencing data have been submitted to the NCBI repositories. The raw sequencing data are available via Sequence Read Archive (SRA) under the BioProject ID—PRJNA1379593. Detailed sample information, including BioSample accession numbers and SRA run accession numbers, is included in Table S5.

### Mutation analysis

To identify mutations in strains, the Illumina DNA sequence was aligned to the genome with bwa-mem2 v2.2.1 (76) and converted to a sorted BAM format alignment file with samtools v1.19.2 (77) and optical duplicates marked with MarkDuplicates in the Picard Toolkit (“Picard Toolkit” 2019. Broad Institute, GitHub Repository. https://broadinstitute.github.io/picard/; Broad Institute). Variants were identified using GATK HaplotypeCaller and GenotypeGVCFs v4.6.0.0 (78). To identify the impact of variants, snpEff v4.3t (79) was run using the AF293 genome and annotation downloaded from FungiDB v68 (67). The resulting VCF file was processed with a custom Python script to generate a table of variants to examine as a spreadsheet. Scripts for processing data and VCF variant call are archived in a GitHub Repository https://github.com/stajichlab/CramerLab_Afumigatus_AF293_VOR and archived in Zenodo at doi: 10.5281/zenodo.17981730.

### Statistics

Unless otherwise stated, all statistics and graphs are generated using GraphPad Prism (v10.6.1) from three biologically independent experiments (unless otherwise stated) with multiple technical replicates in each experiment.

## Data Availability

All data are included in the main article or in the supplemental figures; supplemental tables.

## SUPPLEMENTAL MATERIAL

Supplemental Figures mbio.00661-26-s0001.pdf Figures S1–S6.

Supplemental Tables mbio.00661-26-s0002.xlsx Tables S1–S5.

ASM does not own the copyrights to Supplemental Material that may be linked to, or accessed through, an article. The authors have granted ASM a non-exclusive, world-wide license to publish the Supplemental Material files. Please contact the corresponding author directly for reuse.
