# Supplementary material for: An epigenetic mechanism of azole tolerance facilitates acquired antifungal resistance in Aspergillus fumigatus
Source: mBio. 2026 Jun 15;17(7):e00661-26. doi: 10.1128/mbio.00661-26 (PMC13344070; doi:10.1128/mbio.00661-26)
Supplement: Supplemental Figures — Figures S1–S6. [file mbio.00661-26-s0001.pdf]

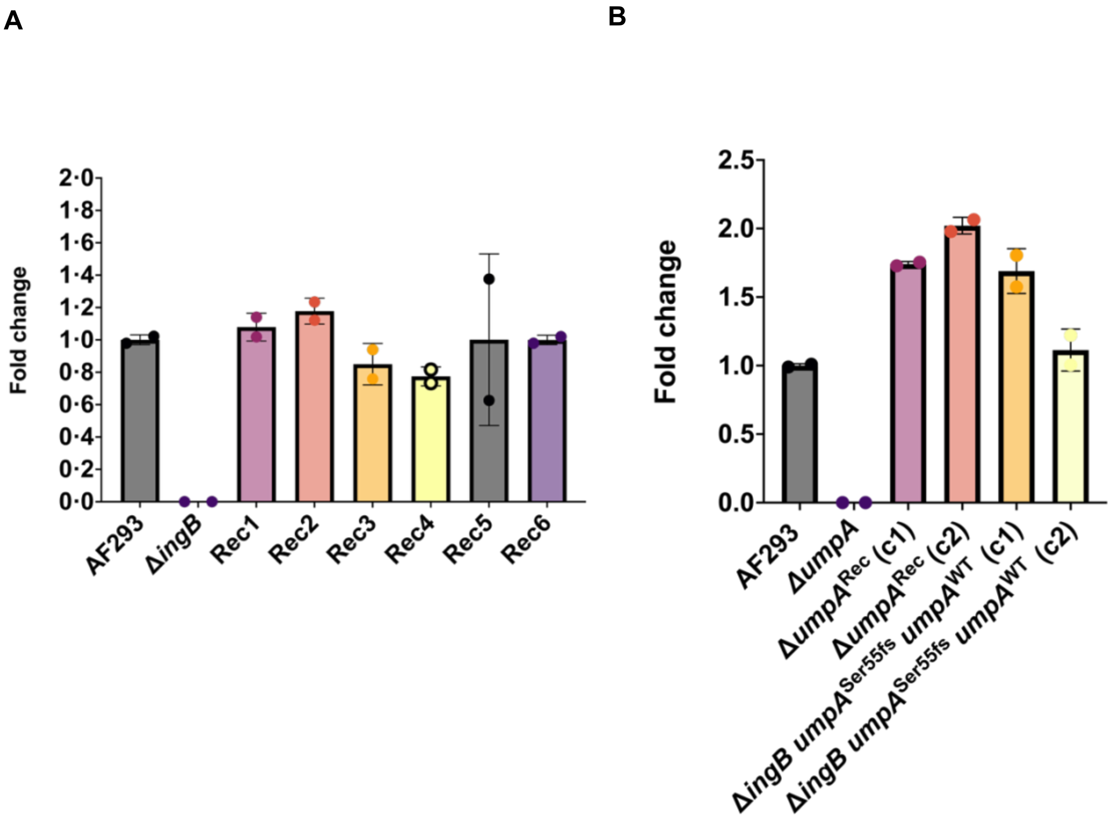

Fig. 1 – Confirmation of loss of expression in the mutant and restoration of expression in the reconstituted strain. A) 10^5 conidia of AF293, mutant, and 6 recon candidates were grown in GMM for 16 hrs at 37 °C, 5% CO_2. RT-qPCR was performed to confirm the restoration of *ingB* gene expression in the recon candidates. *tefA* was used as housekeeping gene. B) 10^5 conidia of AF293, mutant, and 6 recon candidates were grown in GMM for 16 hrs in same conditions. RT-qPCR was performed to confirm the restoration of *umpA* gene expression in recon candidates. *tefA* was used as housekeeping gene. For 1A and 1B each data point represent technical replicates.

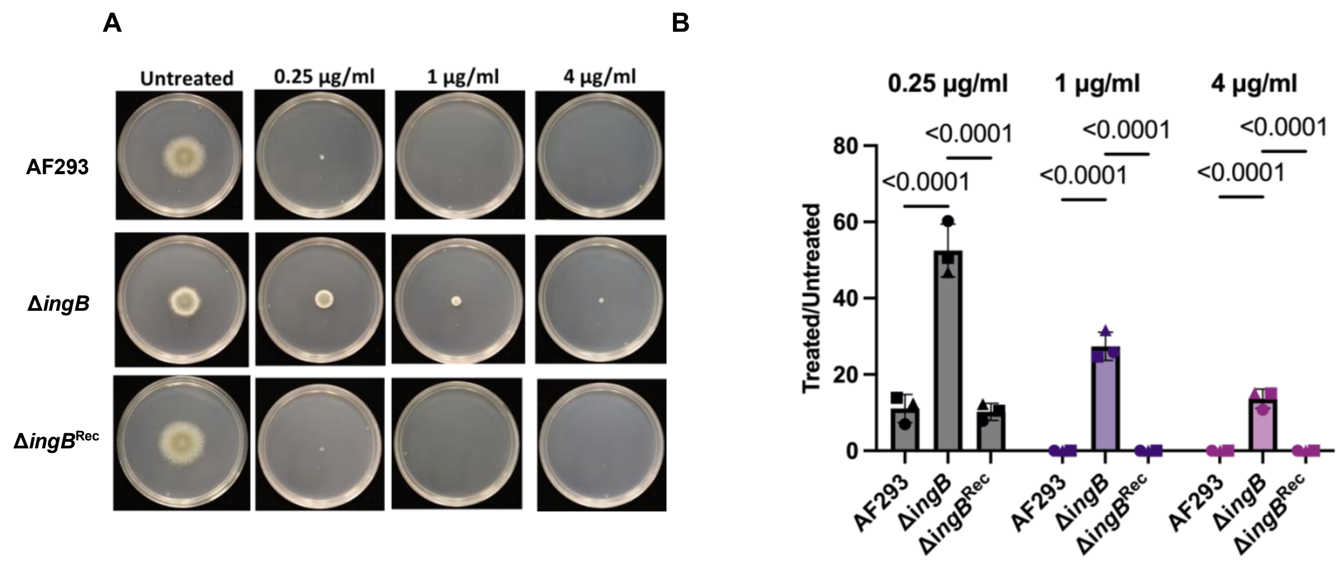

Fig. 2 - Loss of *ingB* results in tolerance to itraconazole. A) 1000 conidia of each strain were spotted in the center of each plate. After 72 hrs of growth at 37 °C, 5% CO2, the radial growth was measured and images were taken. B) Treated to untreated ratios were calculated for each strain. Two-way ANOVA was significant (*$p<0.05$). Dunnett's multiple comparison was used to compare each group with the mutant at all tested concentrations. The p-value is indicated on the graph.

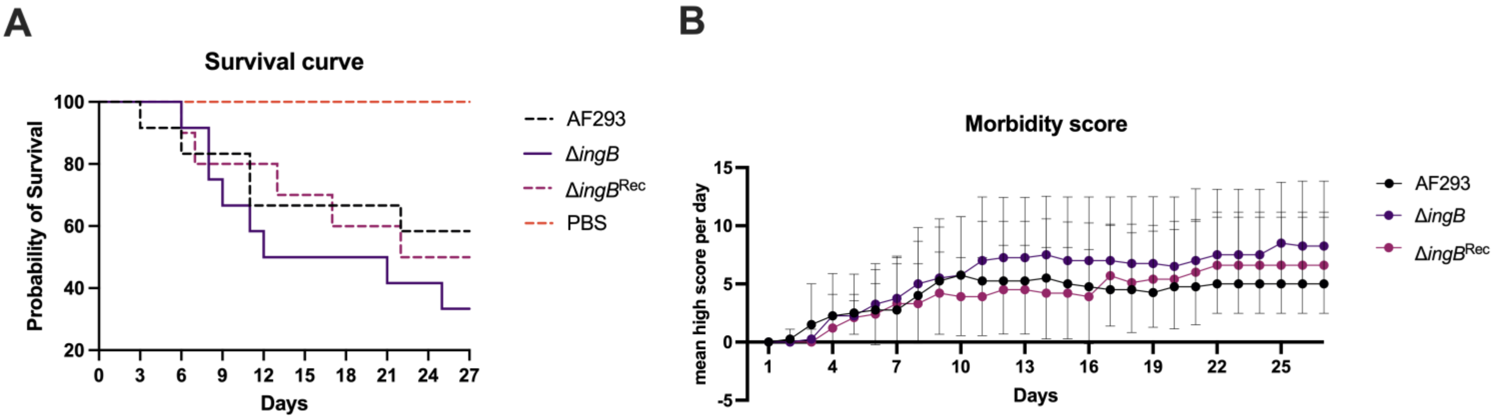

Fig. 3 – Loss of *ingB* does not alter virulence in triamcinolone model of invasive aspergillosis. A) 4–6-week CD-1 female mice were treated with Kenalog-10 (40 mg/kg) one day before inoculation and were infected 10^5 conidia of each strain intranasally. Mock groups were given PBS instead. Mice were monitored for survival. Log-Rank test was not significant (P=0.20; n=10 for mutant and recon, n=12 for wt, and n=4 for PBS). B) Morbidity score for each group was calculated by taking the average of highest score of each mouse in a day. The criteria for scoring system – ruffled – 3 points, hunched – 3 points, reduced activity – 6 points. When the mice reached a score of 12, they were euthanized. Two-way ANOVA was time x column factor was not significant (p=0.30).

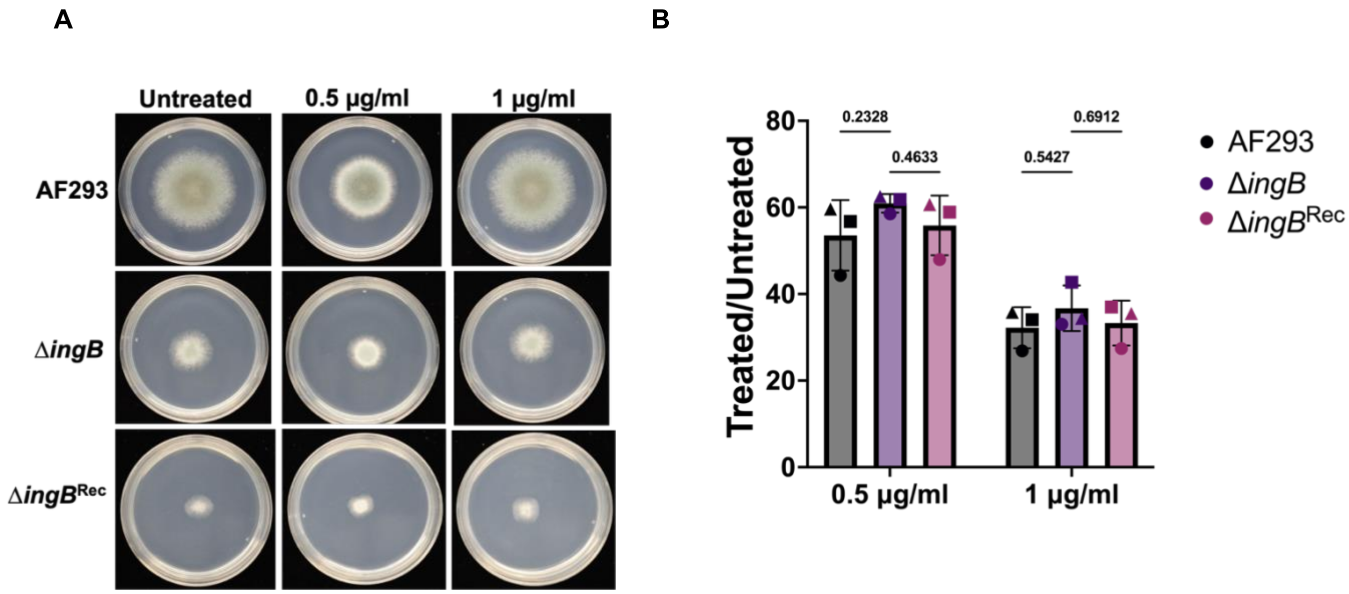

Fig. 4 – Susceptibility to Amphotericin B remains unaltered in ∆*ingB*. A) 1000 conidia of each strain were inoculated in GMM alone or in the presence of Amphotericin B for 72 hrs at 37 °C, 5% CO2. Radial growth was measured and images were taken. The images shown are representative of 3 bioreps. B) Quantification of A. Two-way ANOVA with Dunnet's multiple comparison was performed to compare each group with the mutant, as expected, no significance was observed. The p values are indicated on the graph.

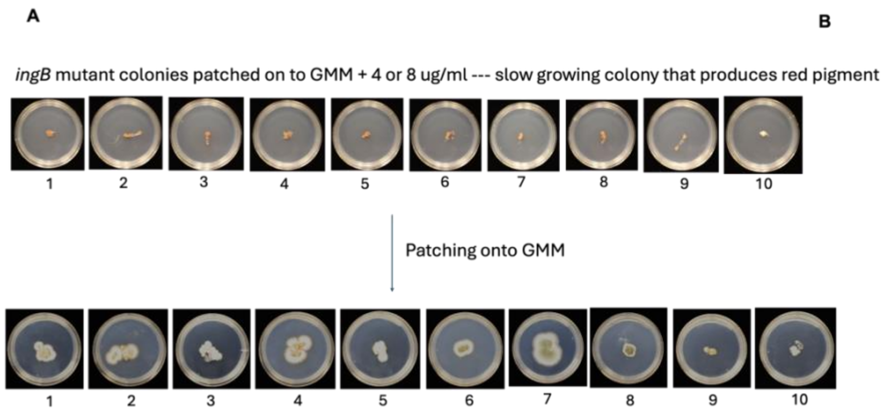

B

Strain	MICs (ug/ml)
AF293	0.25
Δ*ingB*	0.5
Δ*ingB*^{Rec}	0.25
Colony 1	2
Colony 2	2
Colony 3	2
Colony 4	2
Colony 5	2
Colony 6	2
Colony 7	0.5-1
Colony 8	2
Colony 9	2
Colony 10	2-4

Fig. 5 – Morphology of isolates selected on high concentrations of voriconazole. A) A total of 11 colonies were picked and patched onto fresh GMM containing voriconazole. These colonies are characterized by slow growth and production of red pigment. After one week, the colonies were transferred to fresh GMM, and were tested for voriconazole susceptibility using CLSI method. B) The colonies 1, 4, 7, 9, and 11 were sequenced. All isolates selected were resistant to voriconazole. Images and MIC of colony 11 is in Figure 4.

Sequence	Organism	Chromosome	#Genes	Length	Gene Locations
mito_A_fumigatus_Af293	*Aspergillus fumigatus Af293*	N/A	1	31765	
Chr1_A_fumigatus_Af293	*Aspergillus fumigatus Af293*	1	79	4918979	
Chr2_A_fumigatus_Af293	*Aspergillus fumigatus Af293*	2	86	4844372	
Chr3_A_fumigatus_Af293	*Aspergillus fumigatus Af293*	3	80	4079167	
Chr4_A_fumigatus_Af293	*Aspergillus fumigatus Af293*	4	68	3927224	
Chr5_A_fumigatus_Af293	*Aspergillus fumigatus Af293*	5	70	3948441	
Chr6_A_fumigatus_Af293	*Aspergillus fumigatus Af293*	6	68	3778736	
Chr7_A_fumigatus_Af293	*Aspergillus fumigatus Af293*	7	49	2058334	
Chr8_A_fumigatus_Af293	*Aspergillus fumigatus Af293*	8	35	1833124	

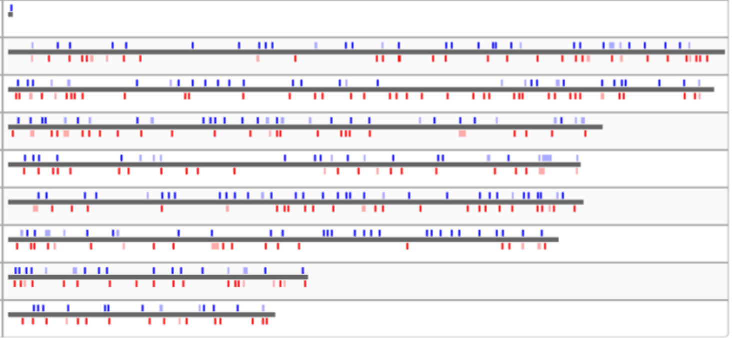

Fig. 6 – IngB acts as a global regulator of transcription. Chromosomal mapping in **FungiDB** (based on **Table S2**) reveals distinct clusters of genes regulated by IngB.
